# Epidemiology of constipation in Europe and Oceania: a systematic review

**DOI:** 10.1186/1471-230X-8-5

**Published:** 2008-02-12

**Authors:** George Peppas, Vangelis G Alexiou, Eleni Mourtzoukou, Matthew E Falagas

**Affiliations:** 1 Alfa Institute of Biomedical Sciences (AIBS), Athens, Greece; 2 Department of Medicine, Tufts University School of Medicine, Boston, Massachusetts, USA

## Abstract

**Background:**

We aimed to review the literature regarding the epidemiology of constipation in Europe and Oceania and the associated prevalence/risk factors.

**Methods:**

Two reviewers performed PubMed searches and a hand search of references. A study was considered eligible for inclusion if it reported data about the prevalence of constipation in any population, free of other gastrointestinal disorders, in Europe and Oceania. Studies were evaluated for quality. Data regarding the setting, type of study, definition of constipation, study population, prevalence of constipation, factors associated with increased odds for constipation, and the female to male ratio, were collected.

**Results:**

The 21 reviewed studies depict prevalence rates in 34 different population groups ranging widely from a low 0.7% to a high 81%. In the general population of Europe the mean value of the reported constipation rates is 17,1 % and the median value 16.6%. Among the studies conducted in Oceania, the mean value of constipation prevalence was 15.3%. Female gender, age and socioeconomic and educational class seem to have major effect on constipation prevalence. A number of various other risk factors are, less clearly, associated with constipation.

**Conclusion:**

This systematic review depicts the high prevalence and related risk factors of a disorder that decreases the health-related quality of life and has major economic consequences.

## Background

Gastrointestinal functional disorders and especially constipation are common morbidity factors in otherwise healthy persons as well as in patients with various predisposing diseases. In the general population, constipation is frequently reported, resulting in 2.5 millions of physician visits in the United States [1] and a total health care cost of $2752 per patient treated [2]. The high prevalence rates, economic cost and adverse implications on the quality of life and health state [3,4], make constipation a major public health issue.

Constipation is due to organic etiologies, such as mechanical obstruction, spinal cord injury [5], stroke, Parkinson's disease, hypothyroidism, diabetes [6] and local neurogenic disorders, e.g. Hirschsprung's disease [7] only for a minority of patients. The majority of cases are attributed to functional disorders without a structural underlying cause that could explain symptoms. Risk factors, such as dietary habits, physical inactivity [8], socioeconomic level, psychological parameters, medications [9], age [10], gender [11], etc have been implicated in the development of chronic functional constipation. However, the specific etiology of this gastrointestinal disorder has not been elucidated.

Only a small proportion of patients suffering from constipation seek medical advice; thus, the exact prevalence of the disorder is difficult to be estimated. A systematic review of studies on the epidemiology of constipation in North America [12] recorded various prevalence rates from a low 1.9% to a high 27.2%. However, most of the included studies, report rates between 12% and 19%. To our knowledge, no previous report of the current evidence has systematically reviewed the epidemiology of constipation in Europe or Oceania.

We sought to review the literature regarding the epidemiology of functional constipation in Europe and Oceania. Specifically, our purpose was to identify the prevalence of constipation in the general population and in special population groups, identify the risk factors associated with this functional disorder and compare the findings with those of the systematic review of the epidemiology of constipation in North America [12].

## Methods

We performed a systematic review of the current evidence regarding the prevalence of constipation in Europe and Oceania. Two reviewers (EGM and VGA) having a medical degree searched the PubMed database (until December 2006) to identify relevant studies for inclusion. The key word used in the literature search was "constipation" combined with "incidence, prevalence, rate, proportion, epidemiology, or morbidity". Studies were limited to those written in English, French and referring to humans. The titles and abstracts of all studies identified were further reviewed to meet the entry criteria. Moreover, we performed a hand search of the references of the selected studies to identify potentially overlooked articles.

A study was considered eligible for inclusion if it reported data about the prevalence of constipation in any population, free of other gastrointestinal disorders, in Europe and Oceania. All studies involving cases of functional constipation, regardless the diagnostic criteria used were considered for inclusion. The diagnostic criteria range widely from self-report and parent-report for children, to consensus criteria like Rome II criteria [13]; at least 12 weeks, which need not be consecutive, in the preceding 12 months, of two or more of: < 3 defecations per week (DPW), hard or lumpy stools in > 1/4 of defecations, straining in > 1/4, digital manipulation of stools in > 1/4, sensation of incomplete evacuation in > 1/4, sensation of anorectal obstruction or blockage in > 1/4.

Two of the authors (EM and VGA) retrieved and studied in detail the complete texts of the original articles that were relevant to the focus of our review. Agreement between the investigators was essential for data inclusion and few minor disagreements were resolved with the consent of the senior author. Data regarding the first author, year of publication, setting, type of study, definition of constipation, study population, prevalence of constipation, factors associated with increased odds for constipation, and the female to male ratio, were extracted. The above were tabulated in detail without taking into consideration the differences in methodology among the reviewed studies. We calculated the mean values for prevalence of constipation in Europe and Oceania by averaging the prevalence obtained in each study.

We used the critical appraisal guidelines for research articles determining prevalence, developed by Loney et al [14], to evaluate the quality of studies included in our systematic review. The scoring system consists of 8 questions on the validity of the study design (appropriate methods and frame, adequate size, suitable criteria, outcome measurements, and response rate), the interpretation (prevalence given with confidence intervals) and the applicability of the results (detailed description of subjects and settings), weighted equality with one point [15]. Thus the maximum quality score that a study could achieve was 8.

## Results

PubMed database returned a list of 2060 articles for the selected search string. From these, 2039 reports were excluded, after having reviewed their titles and abstracts, based on the criteria outlined in the Methods section. The full texts of the remaining 21 studies [16-36] were read and finally included in our systematic review. One more study [37] was identified following the hand search of references. However, Galvez et al evaluated a population already described by Garrigues et al study (included in this review). Reviewers disagreed on the inclusion of three studies [25,26,33]. However, they were included with the consent of the senior author.

In Tables 1, 2 and 3, we summarize the characteristics (author, year, type of study, depicted epidemiological data and quality score) of the reviewed studies regarding the prevalence of constipation in the general population of Europe, in special population groups of Europe and in all population groups of Oceania respectively. Overall the reviewed studies depict prevalence rates in 34 different population groups ranging widely from a low 0.7% in a pediatric population in Italy [30] to a high 81% in a hospitalized, elderly, male population [24]. The mean value of the reported constipation rates is 22.3 and the median value 19.8. More than half of the reported constipation rates range from 8% to 26%.

**Table 1:** Characteristics of studies regarding the epidemiology of constipation in Europe in the general population.

**First author, year of publication, setting**	**Type of study**	**Definition of constipation**	**Study population (% of responders)**	**Prevalence per 100**	**Factors significantly associated with increased odds for constipation**	**Quality score^+^**	**Comments**
Siproudhis L 2005 France [16]	Mailed questionnaire survey	Emptying difficulties or unsatisfied defecation during the preceding 12-month period	7196 (72) persons ≥ 15 yr, representative of the general population	22.4	History of vaginal delivery in women	6	F/M 2
Garrigues V 2004 Spain [17]	Mailed questionnaire study	Self-reported, Rome I, and Rome II criteria	349 (71.4) participants, 18–65 yr, representative of the general population	29.5 (self-reported) 19.2 (Rome I criteria) 14 (Rome II criteria)	Female gender	7	F/M 2.2 Agreement between self-reported and Rome II criteria was moderate
Walter S 2002 Sweden [18]	Mailed questionnaire study	Self-reported, as 'sometimes, often or always' constipated	1610 (80.5) persons, 31–76 yr, randomly selected	19.8 of women 8.3 of men	Female gender	4	F/M 2.4
Haug TT 2002 Norway [19]	Mailed questionnaire study	Self-reported, as 'minor or major complaints' during the last 12 months	62651 (66.5) persons, > 20 yr	20.2	Anxiety, and less strongly depression	4	F/M 4
Frexinos 1998 France [20]	Interview- questionnaire survey	Self reported	6000 (81) participants > 15 yr, selected by systematic random sampling	35		7	
Gaburri 1989 Italy Umbria [21]	Interview-questionnaire survey, performed by a physician.	≤ 2 DPW for > 9 months in the last 3 years and/or straining at stool > 75% of the time	544 (98). 104 medical students, 103 medical and paramedical employees and 326 randomly selected home interviewed persons.	9.2	Female gender presented significantly higher constipation rates.	5	
Bassotti G 2004 Italy [22]	Prospective study	Rome II criteria	298 (61) persons recruited from the general population and asked to compile a daily diary on their bowel habits and associated signs and symptoms	5 (< 3 DPW) 11.7(straining) 10.7(incomplete evacuation)		6	

**Abbreviations **DPW: defecations per week, F/M: female/male ratio, Rome II criteria: at least 12 weeks, which need not be consecutive, in the preceding 12 months, of two or more of: < 3 DPW, hard or lumpy stools in > 1/4 of defecations, straining in > 1/4, digital manipulation of stools in > 1/4, sensation of incomplete evacuation in > 1/4, sensation of anorectal obstruction or blockage in > 1/4, ^+^**: **critical appraisal guidelines for research articles determining prevalence, developed by Loney et al^14^

**Table 2:** Characteristics of studies regarding the epidemiology of constipation in Europe, in special populations groups.

**First author, year of publication, setting**	**Type of study**	**Definition of constipation**	**Study population (% of responders)**	**Prevalence per 100**	**Factors significantly associated with increased odds for constipation**	**Quality score^+^**	**Comments**
Lopez Cara MA 2006 Spain [23]	Mailed questionnaire survey	≤ 3 DPW	414 (93) participants > 50 yr, selected by systematic random sampling	4.4	Consumption of olive oil, and meat.	4	F/M 2
Kinnunen 1990 Finland [24]	Interview-questionnaire survey by public health nurse	≤ 3 DPW, difficulties in expelling stools because of the hardness or anal canal abnormalities	5 groups of middle aged and elder population. 1. Hospital: 439 2. Olds people's home: 183 3. Day hospital: 78 4. Home > 74 yrs: 138 5. Home 41–50 yrs: 74	Prevalence per 100 in the 5 groups 1st: females 79, males 81 2nd: females 57, males 64 3rd: females 30, males 25 4th: females 38, males 37 5th: females 20, males 3	Female gender. Fecal and urinary incontinence. Age. Immobility. Living in old people's homes and geriatric hospital. Age over 84 years.	5	
Bommelaer 1986 France [25]	Interview-questionnaire survey, performed by a physician.	≤ 3 DPW	1200 participants. Randomized selection among healthy medical and para-medical personnel and medical students. Statistically tested to assure same participation of gender, age and socio-professional groups	6.3	Female gender. Use of laxatives.	3	F/M 1.12
Texerau 1989 France [26]	Interview-questionnaire survey, performed by a gastroenterologist.	Self reported	667 participants in 4 groups. 82 medical students, 206 patients from local health center, 210 adults interrogated in an occupational medicine office and 69 persons from an olds people house.	26		3	F/M 0.96
Chin A Paw M 2006 The Netherlands [27]	RCT investigating the effects of training on constipation, questionnaire study	Defined below*	172 (76.8) participants living in long-term care facilities, 64–94 yr	22		5	
Ludvigsson JF 2006 Sweden [28]	Prospective cohort study, questionnaire survey	Reported by parents	8341 (38.4) 2.5-yr-children from a birth cohort	6.5	Low maternal education, female sex, living in a large community, lack of older siblings	3	
Iacono G 2005 Italy [29]	Prospective study, data obtained from 150 paediatricians	One bowel movement every 3 days or more	2879 (96) newborns up to six months of age	17.6	Lower frequency of breastfeeding.	4	F/M 1
Miele E 2004 Italy [30]	Prospective study, data obtained from 13 randomly selected paediatricians	Rome criteria for children	9660 children, 0–12 yr	0.7		5	F/M 1.2
Soligo M 2006 Italy [31]	Retrospective survey	Decreased stool frequency, difficult stool passage	786 consecutive urogynecologic patients, average age 60 yr	31.7	Posterior colpocele	4	

**Abbreviations **DPW: defecations per week, F/M: female/male ratio, RCT: randomized controlled trial, Rome criteria for children: in infants and preschool children, at least 2 wk of pebble-like, hard stools for a majority of stools, firm stools 2 or fewer times per week, and no evidence of structural, endocrine, or metabolic disease, *: at least one of less than three bowel movements weekly, hard or lumpy stools, straining on defecation, incomplete evacuation, in the preceding 12 months ^+^**: **critical appraisal guidelines for research articles determining prevalence, developed by Loney et al^14^

**Table 3:** Characteristics of studies regarding the epidemiology of constipation in Oceania.

**First author, year of publication, setting**	**Type of study**	**Definition of constipation**	**Study population (% of responders)**	**Prevalence per 100**	**Factors significantly associated with increased odds for constipation**	**Quality score^+^**	**Comments**
Howell SC 2006 Sydney [32]	Mailed questionnaire survey	Rome II criteria	1673 (42.6) persons, 25–64 yr, randomly selected from 28 districts	30.7	Upper-middle educational social class. Female gender.	6	F/M 1.4
Campbell 1992 New Zealand [33]	Interview-questionnaire survey and a dietary assessment	≤ 3 DPW, strained at stools, taking laxatives every 2–3 days	856 (91) participants> 70 years registered with the five general practitioners serving a rural township of 13500 people	4.3 had ≤ 3 DPW 20.3 had one of the three factors that may indicate constipation	Female gender and age	3	F/M 1.76
Bytzer P 2001 Sydney [34]	Mailed questionnaire survey	At least one of < 3 DPW, hard or lumpy stools, anal blockage, during preceding 3 months	8555 (57), divided into 5 socioeconomic classes, from 1^st ^(highest) to 5^th ^(lowest) quintiles	1^st^: 6.3 2^nd^: 8.7 3^rd^: 9.6 4^th^: 10.3 5^th^: 10.2	Low socioeconomic class.	6	
Chiarelli P 2000 Australia [35]	Questionnaire survey	Sometimes or often experiencing constipation symptoms during the preceding 12 months	14761 (41) women 18–23 yr 14070 (54) women 45–50 yr 12893 (37) women 70–75 yr	14.1 (18–23 yr) 26.6 (45–50 yr) 27.7 (70–75 yr)	Haemorrhoids and 'other bowel problems' in all three cohorts. Parity in the young cohort. Hysterectomy, prolapse repair, and medications in the middle-aged and older cohorts.	3	
Talley NJ 2004 New Zealand [36]	Questionnaire survey	Defined below*	924 persons 26 yr old, from a birth cohort (94 of total original sample)	19.9	Female gender.	5	F/M 1.3

**Abbreviations **DPW: defecations per week, F/M: female/male ratio, Rome II criteria: at least 12 weeks, which need not be consecutive, in the preceding 12 months, of two or more of: < 3 DPW, hard or lumpy stools in > 1/4 of defecations, straining in > 1/4, digital manipulation of stools in > 1/4, sensation of incomplete evacuation in > 1/4, sensation of anorectal obstruction or blockage in > 1/4, *: at least one of less than three bowel movements weekly, hard or lumpy stools, straining on defecation, incomplete evacuation, in the preceding 12 months, RCT: randomized controlled trial, ^+^**: **critical appraisal guidelines for research articles determining prevalence, developed by Loney et al^14^

Seven studies have data on the prevalence of constipation in the general population in Europe (Table 1). The reported constipation rates range from a low 5% in a prospective study using the diagnostic criterion of < 3 defecations per week (DPW) [22] to a high 35% in an interview questionnaire survey of self-reported constipation [20]. The mean value of the reported constipation rates is 17.1 % and the median value 16.6%. Nine studies depict constipation prevalence rates in special population groups in Europe: infants, children, elderly population, women and participants living in long-term care facilities (Table 2). Finally, 5 studies regarding the epidemiology of constipation in Oceania are tabulated in Table 3. The lowest prevalence was observed among an elderly population in New Zealand (4.3%) [33]. Howel et al recorded the highest prevalence rate of constipation in the general population of Sydney Australia (30.7%). Among the studies conducted in Oceania, the mean value of constipation prevalence is 15.3%.

A secondary purpose of this review was to identify risk factors related with this functional constipation disorder. In particular, the predominance of females in the constipation prevalence is documented in most of the reviewed studies. In order to depict the exact magnitude of this association we present in Tables 1, 2 and 3 the female/male ratio, in the studies where available. The mean and median value of the above ratio is calculated to 1.78 and 1.58 respectively. There are differences of the female/male ratio estimates by definition of constipation. However, the mean value remains above 1 for all case criteria, 1.7 for Rome I, 1.8 for Rome II, and 2.3 for self-report of constipation.

The effect of ageing on constipation prevalence has been recorded by 3 of the reviewed studies [24,27,33]. Socioeconomic and educational class influence on the constipation prevalence is also depicted in 3 studies [28,32,34]. Various other risk factors are associated with constipation by at least one research paper included in our systematic review of the current evidence; dietary habits like the consumption of olive oil and meat, life-style factors like living in a large community or old peoples home and immobility, frequency of breastfeeding, waist/hip ratio, anxiety and depression, co morbidities like hemorrhoids, other bowel disorders, previous hysterectomy and posterior colpocele and the use of medications like laxatives.

## Discussion

The prevalence rates of constipation in Europe and Oceania as depicted by this review are consistent with the epidemiology of the disorder in North America [12]. Prevalence rates of constipation recorded in other developed countries are also within the same range; 14.3% in the general population in Hong-Kong [38], 16.5% in the general population in Korea [39], 24.5% in women in Taiwan [40], 26% in a population of young women in Japan [41], 29.6% in young children in Hong Kong [42], and 11.6% in an elderly Asian population [43]. The similarity of constipation rates recorded in the developed countries may partially be due to the common dietary habits of the studied populations. Constipation in individuals with previously normal bowel function is associated with specific dietary patterns; low fiber intake diet is involved with the pathogenesis of the disorder. Europe and Oceania are similar to North America in terms of health system and population's dietary habits, life style, physical activity and socioeconomic level.

Differing case definitions of constipation used, provide researchers with diverse prevalence rates. This is evident in the Garrigues et al study, where different diagnostic criteria were compared in the same individuals, resulting in significant differences in the depicted prevalence rates; 29.5% for self-report, 19.2% for Rome I criteria and 14% for Rome II criteria. We identified 3 studies using the Rome II criteria; the mean prevalence rate among these reports is 16.5%. The relevant mean prevalence rates for Rome I criteria and self-report are 19.2% and 25.6% respectively. It should be mentioned that the Rome III consensus provides the most up to date definition and categorization of functional gastrointestinal disorders in children/adolescent [44] and neonate/toddler [45]. None of the reviewed studies implemented the Rome III criteria. Future studies on the epidemiology of constipation should rather be based on consensus diagnostic criteria.

Female gender is associated with elevated constipation prevalence rates [17,18,21,25,32,33,36]. In North America, females are 2.2 times more likely to report constipation than males [12]. This predominance of females has been attributed to hormonal factors, inflicting a higher risk of constipation during the luteal phase of the menstrual cycle, under the effect of progesterone, and damage to the pelvic floor muscles, which may occur in women during childbirth or gynecological surgery [35]. Posterior colpocele is an independent risk factor for constipation [31]; however, causal relationship between these disorders is difficult to be assumed.

In general, individuals of lower social, economic and educational level have a tendency towards higher constipation rates. Bytzer et al divided the sample of their questionnaire survey into five socioeconomic classes from 1^st ^(highest) to 5^th ^(lowest). They showed that the constipation prevalence rates among those five groups followed the pattern of their socioeconomic class, ranging from a low 6.3% for the 1^st ^class to a considerably higher 10.2% for the 5^th ^class. Of interest, according to another study [28], low maternal educational level is considered as a factor significantly associated with increased odds for constipation of the newborn.

Chiarelli et al questionnaire survey compares constipation prevalence among three age groups, 18–23 years, 45–50 years and 70–75 years and depicts considerable differences in prevalence rates; 14.1%, 26.6% and 27.7% respectively. A study focusing on infants followed up for 6 months, showed a prevalence rate of 17.6% [29]. This is surprisingly high when compared with the constipation prevalence in a pediatric population aged from 0–12 years [30]; Miele et al used the Rome criteria in 9960 children and report a prevalence rate of 0.7%.

Psychological factors and in particular anxiety and depression, are considered predisposing to constipation [19]. These psychological disorders, as well as obsessive compulsion and social dysfunction, have been implicated in the pathogenesis of constipation, are believed to slow down colonic transit, have greater impact on women, and are associated with less frequent use of coping strategies [38]. Behavioral treatment, specifically biofeedback, is alleged to ameliorate the course of the disorder and is frequently used as alternative therapy for persons with symptoms unresponsive to traditional medical treatment [46,47].

Assessing quality in clinical trials is well described but much less attention has been given to similar strategies for observational epidemiological studies. We believe that the quality assessment tool used [14,15] addresses efficiently the need for critical appraisal of prevalence studies. There were significant differences in the quality and methodology among the reviewed studies. The best score achieved was 7/8 and the worst 3/8 (Tables 1, 2 and 3).

It should be acknowledge that when interpreting the findings of this systematic review one should take under consideration various limitations and shortcomings. First, the included studies use different definitions and diagnostic criteria of constipation, which could lead to inconsistencies regarding the prevalence estimates. No doubt, there is a discrepancy between self-reported constipation and the condition diagnosed based on the established Rome criteria. The rates are considerably higher when based on the definition of self-reported constipation [26]. This discrepancy highlighted by other investigators too [39], may be due to differences of personal perception regarding the problem, or could be involved with the validity of criteria. In addition, the use of questionnaires depends on the ability of the patient to recall symptoms, whereas prospective studies with the administration of diary cards are certainly more credible. Furthermore, the range of age groups studied is wide, and includes pediatric, as well as elderly study populations. Finally, it should be acknowledged that our search of the literature was limited to journals included in the PubMed database. However, the further hand search of references reduces possibly omitted studies. The language limit (articles published in English and French only) should be attributed to the available human resources. Despite this limitation, studies from 7 different European countries were included in our review (Spain, France, Italy, Netherlands, Norway, Sweden and Finland).

## Conclusion

Because of the decrement in the health-related quality of life induced by constipation, and the economic consequences, physicians should be aware of the magnitude of the problem, and be able to give efficient instructions to patients for management, and particularly, prevention. We believe that our systematic review of current evidence on constipation provides a useful tool to physicians dealing with this high prevalence clinical problem, and a reference for future development of studies on the epidemiology and etiology of the disorder.

## Competing interests

The author(s) declare that they have no competing interests.

## Authors' contributions

GP conceived of the study, GP and MEF participated in its design and coordination. VGA, EM and MEF searched the literature and drafted the manuscript. All authors read and approved the final manuscript.

## Pre-publication history

The pre-publication history for this paper can be accessed here:


